# Stiff wire loop technique for the management of jailed iliac vein stents in a patient with phlegmasia

**DOI:** 10.1016/j.jvscit.2025.101853

**Published:** 2025-05-23

**Authors:** Paulina Villanueva, Kyongjune Benjamin Lee, Bryan W. Tillman

**Affiliations:** Division of Vascular Diseases and Surgery, Department of Surgery, The Ohio State University Wexner Medical Center, Columbus, OH

**Keywords:** Deep vein, Misplaced stents, Phlegmasia, Stent migration, Stent thrombosis, Thrombosis

## Abstract

Obtaining wire access around a jailed vascular stent remains a difficult challenge for endovascular interventions. We present the case of a 79-year-old with phlegmasia cerulea dolens after remote jailed iliac vein stent amidst acute deep venous thrombosis with phlegmasia. After repeated unwanted passage of a soft guidewire into interstices of the bare metal stent, a stiff wire loop was instead used to pass the wire outside of the stent, with subsequent parallel stenting and restoration of venous outflow.

A barrier to the management of misplaced bare metal stents is navigating a wire without entangling the stent interstices. We report on a 79-year-old with phlegmasia cerulea dolens after iliac vein stenting. Computed tomography (CT) imaging revealed misplacement of the proximal stent into the hypogastric vein, jailing the external iliac vein, prohibitive to restoring flow. After repeated wire entry through stent struts, a loop was created with a stiff wire to promote wire passage alongside the stent. Once wire access was accomplished, a secondary stent was placed in a kissing stent format. This technique may have applications to other misplaced stent scenarios.

## Case report

**Fig 1:**
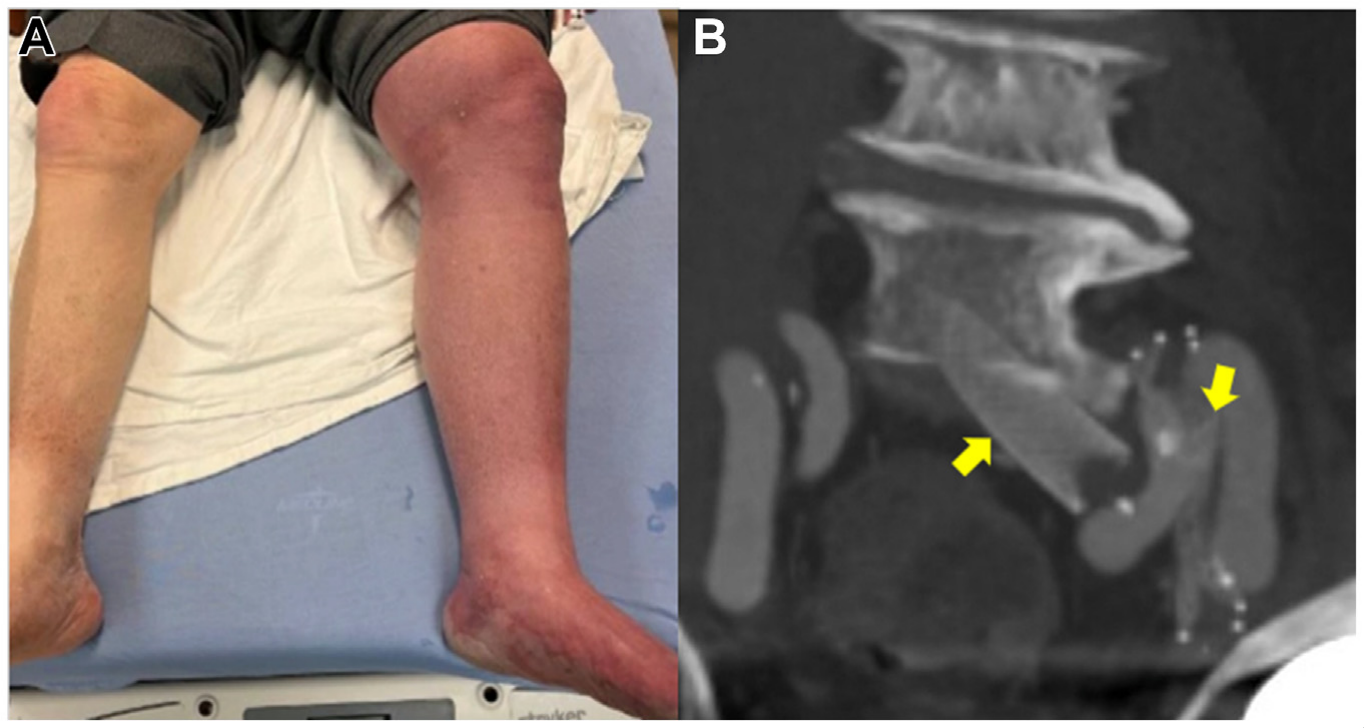
**(A)** Patient presentation with left leg phlegmasia cerulea dolens. **(B)** Computed tomography (CT) imaging revealed the two previous iliac stents in discontinuity.

**Fig 2:**
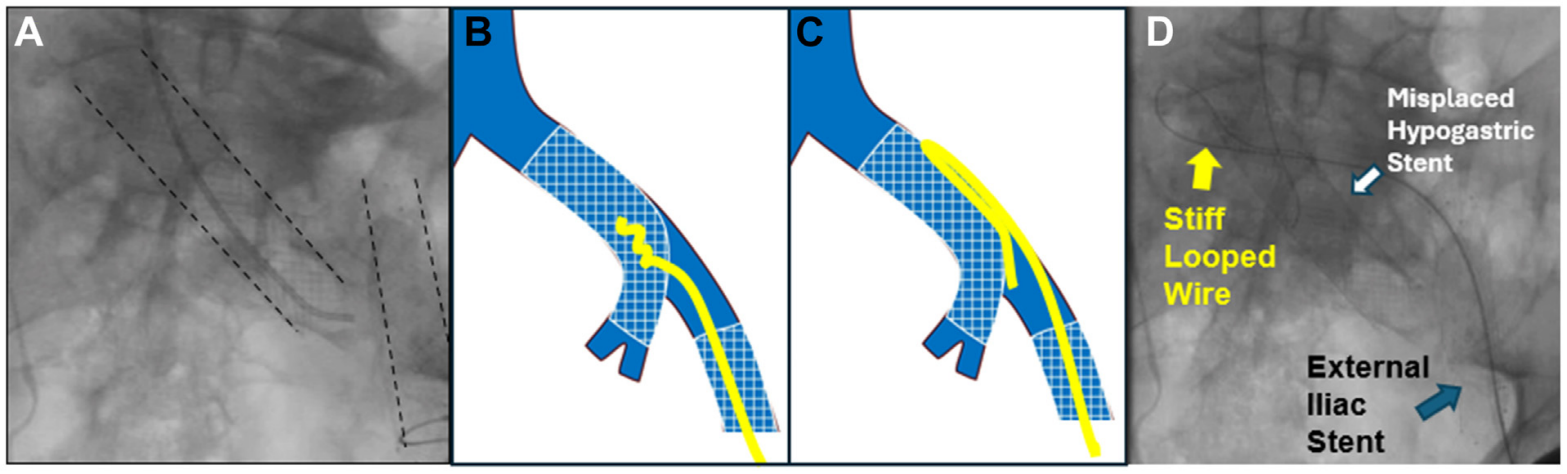
**(A)** Reveals the displaced bare metal common iliac stent into the hypogastric and discontinuity form the external iliac stent (*dashed lines outline original stents*). **(B)** A glidewire became entangled within stent struts. **(C)** Using a loop from a stiffer wire allowed passage outside the stent. **(D)** Fluoroscopic image of the stiff looped wire passing outside the hypogastric stent.

**Fig 3:**
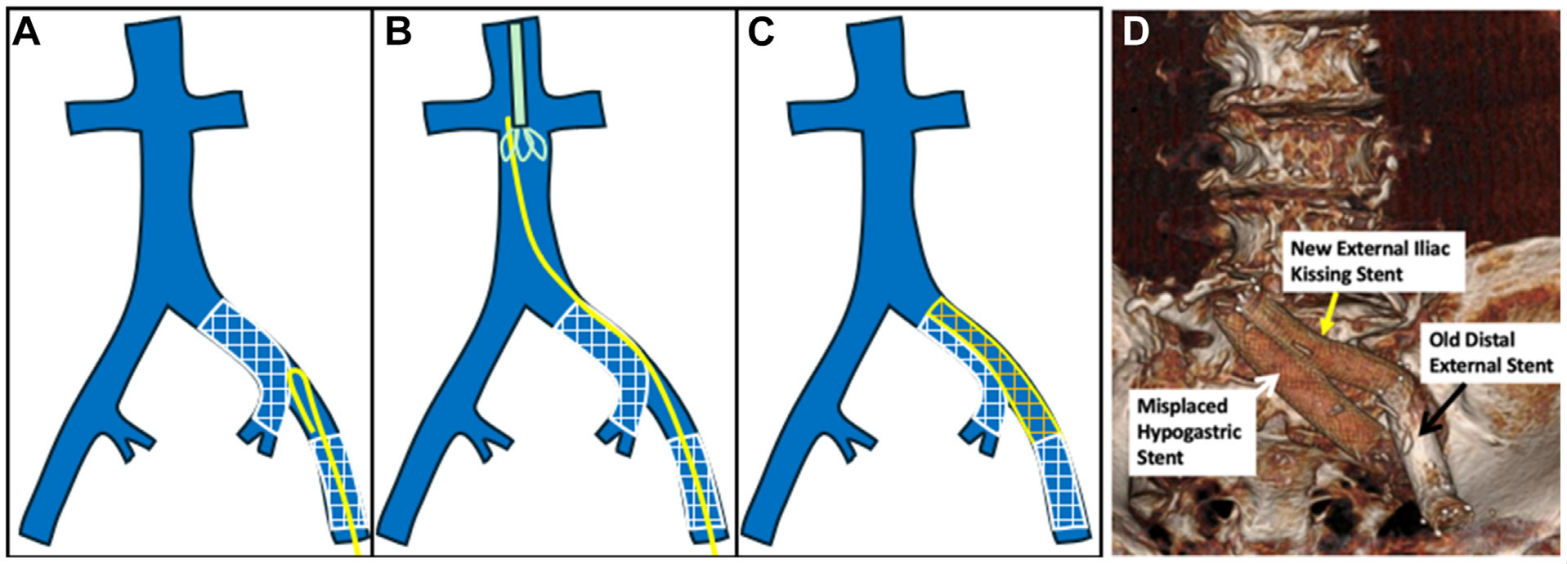
**(A)** Following passage of a stiff looped wire past the misplaced stent, the wire was snared in the inferior vena cava (IVC) **(B)** for placement of a secondary kissing stent graft **(C** and **D)**.

A 79-year-old male with past medical history of May-Thurner syndrome presented with phlegmasia cerulea dolens of his left lower extremity (Fig 1, *A*). His past surgical history was significant for bare metal stents in the left common iliac vein (CIV) and a separate stent in the external iliac vein (EIV) 12 years prior to presentation. The patient reported that 8 days prior, he discontinued long-term anticoagulation due to recent onset of hematuria. A CT venogram revealed thrombosis of his external iliac vein stent but, of more concern, apparent discontinuity of the two iliac stents (Fig 1, *B*). The patient was taken to the operating room for intended venogram, mechanical thrombectomy, and decompressive fasciotomies. Discontinuity of the stents was validated venographically (Fig 2, *A*). The first stent was placed from the CIV into the internal iliac vein (IIV), and a separate stent was placed in the EIV. The CIV stent was a Wallstent (Boston Scientific), 18 mm × 60 mm. The EIV stent was a S.M.A.R.T (Cortis) stent, 14 mm × 60 mm. Several attempts were made to navigate a Glidewire (Terumo Interventional Systems) outside the misplaced proximal stent, yet the wire repeatedly prolapsed through the stent interstices instead (Fig 2, *B*). Given the pressing need to restore outflow for this limb, we instead employed a Meier J-tip wire (Boston Scientific) from a femoral approach, creating a large stiff loop that easily pushed outside the proximal stent (Fig 2, *C* and *D*, and 3, *A*) and into the vena cava. The wire was then snared from a jugular approach to achieve a “body-floss” technique (Fig 3, *B*). A 20 F Protrieve Sheath (Inari Medical) was placed from the right jugular vein into the inferior vena cava to prevent intra-procedural pulmonary embolism. We decided that instead of crushing the original Wallstent, we would instead place a new stent in a kissing format to preserve flow from both the hypogastric vein and external iliac vein. We used a 13 mm × 10 cm Viabahn stent (Gore Medical) followed by a 13 mm × 5 mm Viabahn stent, which we placed from the CIV to the EIV. The stents were sized using the preoperative CT scan without the use of intravascular ultrasound. A covered self-expanding stent was selected to help appose any clot burden to the vessel wall and help prevent any further vessel occlusion (Fig 3, *C* and *D*). Although Viabahn stents are generally not large enough to use for deep venous work, using the CT venogram, we determined that the distal EIV measured approximately 10 mm. We concluded that the 13-mm Viabahn would provide adequate seal. Stents were post-dilated with a 12-mm Atlas balloon (Becton Dickinson). Mechanical thrombectomy of the EIV and femoral veins was completed with a FlowTriever 16F (Inari Medical) catheter, providing immediate restoration of flow and resolution of phlegmasia changes prior to four-compartment calf fasciotomies. The following day, the patient underwent primary closure of fasciotomy incisions. Postoperatively, following a urologic workup for hematuria and resumption of anticoagulation, the patient was discharged. At follow-up, the patient was noted to have well-healed fasciotomies, and he was tolerating both compression and anticoagulation. After discussion with the patient, consent for publication was obtained.

## Discussion

Stent misplacement, whether at the time of the initial placement or by chronic migration, remains a challenging problem. By reviewing the patient’s initial venogram images, we confirmed that the two stents were initially placed in discontinuity. The first stent was placed from the CIV into the IIV, and a separate stent was placed in the EIV. Misplacement was likely contributory to his deep vein thrombosis and phlegmasia. As a result, meaningful restoration of the venous channel was crucial. Because the patient presented with limb-threatening phlegmasia, we did not believe that thrombectomy of the misplaced stent from the CIV to hypogastric would effectively resolve the underlying problem; rather, we elected to stent from the EIV to CIV to restore this critical venous outflow. This technique, in combination with fasciotomies, treated his phlegmasia. The risk of limb loss in patients with phlegmasia cerula dolens has been reported to be 10% to 25%.^1^ If progression to venous gangrene occurs, the rate of limb loss is between 20% and 50% with a mortality rate of 20% to 40%.^1^

Technically, the use of many guidewires with bare metal stents can be challenging, as the wire becomes entangled in the interstices of the stent. Although wire entrapment is a rare complication, it is more likely to occur in total occlusions and in-stent restenotic lesions.^2^

The floppy nature of the Glidewire makes it prone to irregular coiling, which is unhelpful for traversing outside a bare metal stent. In contrast, the stiff nature of the Meier wire allowed creation of a large singular loop that prevented the wire from prolapsing into the interstices of the bare metal stent and facilitated passage outside of the stent.

Other techniques described for the management of thrombosed iliac stents include balloon venoplasty with placement of additional stents and balloon angioplasty through the interstices of the thrombosed stents.^3^ We felt that our reported approach was the best way to ensure flow from both the IIV and EIV and avoid complications such as a fractured stent.

## Conclusions

The report demonstrates the utility of a large stiff wire loop technique to traverse alongside an existing stent without passing through the interstices of the stent and is especially valuable for management of misplaced stents. This technique may also have utility for arterial scenarios.

## Funding

None.

## Disclosures

None.
